# Tumor-targeting *Salmonella typhimurium* A1-R suppressed an imatinib-resistant gastrointestinal stromal tumor with c-kit exon 11 and 17 mutations

**DOI:** 10.1016/j.heliyon.2018.e00643

**Published:** 2018-06-06

**Authors:** Kentaro Miyake, Kei Kawaguchi, Masuyo Miyake, Ming Zhao, Tasuku Kiyuna, Kentaro Igarashi, Zhiying Zhang, Takashi Murakami, Yunfeng Li, Scott D. Nelson, Michael Bouvet, Irmina Elliott, Tara A. Russell, Arun S. Singh, Yukihiko Hiroshima, Masashi Momiyama, Ryusei Matsuyama, Takashi Chishima, Shree Ram Singh, Itaru Endo, Fritz C. Eilber, Robert M. Hoffman

**Affiliations:** a AntiCancer Inc., San Diego, CA, USA; b Department of Surgery, University of California, San Diego, CA, USA; c Department of Gastroenterological Surgery, Yokohama City University Graduate School of Medicine, Yokohama, Japan; d Division of Hematology-Oncology, University of California, Los Angeles, CA, USA; e Deptartment of Pathology, University of California, Los Angeles, CA, USA; f Division of Surgical Oncology, University of California, Los Angeles, CA, USA; g Basic Research Laboratory, National Cancer Institute, Frederick, MD, USA

**Keywords:** Biochemistry, Cancer research, Genetics, Microbiology

## Abstract

Gastrointestinal stromal tumor (GIST) is a refractory disease in need of novel efficacious therapy. The aim of our study was to evaluate the effectiveness of tumor-targeting *Salmonella typhimurium* A1-R (*S. typhimurium* A1-R) using on a patient derived orthotopic xenograft (PDOX) model of imatinib-resistant GIST. The GIST was obtained from a patient with regional recurrence, and implanted in the anterior gastric wall of nude mice. The GIST PDOX mice were randomized into 3 groups of 6 mice each when the tumor volume reached 60 mm^3^: G1, control group; G2, imatinib group (oral administration [p.o.], daily, for 3 weeks); G3, *S. typhimurium* A1-R group (intravenous [i.v.] injection, weekly, for 3 weeks). All mice from each group were sacrificed on day 22. Relative tumor volume was estimated by laparotomy on day 0 and day 22. Body weight of the mouse was evaluated 2 times per week. We found that *S. typhimurium* A1-R significantly reduced tumor growth in contrast to the untreated group (*P* = 0.001). In addition, we found that *S. typhimurium* A1-R was more effective compared to imatinib (*P* = 0.013). Furthermore, Imatinib was not significantly effective compared to the control group (P = 0.462). These results indicate that *S. typhimurium* A1-R may be new effective therapy for imatinib-resistant GIST and therefore a good candidate for clinical development of this disease.

## 1 Introduction

Gastrointestinal stromal tumor (GIST) is a rare and recalcitrant tumor and may originate from the interstitial cell of Cajal [1]. It is estimated that each year the total number of new cases of GIST in the United States will be between 4,000–6,000. Complete tumor excision without tumor rupture is the only known curative therapy of GIST [2]. In most cases, GIST results from a mutation in one of the two tyrosine kinase receptors genes, c-kit (75%, exon: 9, 11, 13 and 17) or platelet-derived growth factor receptor α (PDGFRα, 10%; exon: 12, 14 and 18) [3].

Standard known chemotherapies are not effective against GIST. Three tyrosine kinase inhibitors (TKIs) with c-kit inhibitory activity have been approved for the treatment of GIST (imatinib, sunitinib, and regorafenib). Imatinib, a 2-phenyl amino pyrimidine derivative, is an inhibitor of several tyrosine kinase enzymes [4, 5], and first-line therapy for GIST [5]. Imatinib was first developed as a treatment for chronic myeloid leukemia (CML) by inhibiting the intracellular tyrosine kinase termed the Abelson murine leukemia viral oncogene homolog (ABL) and the kinase breakpoint cluster region protein (BCR)-ABL fusion protein [6, 7]. Imatinib inhibits the downstream signalling cascade, which regulates cell proliferation by interrupting the transfer of phosphate groups from adenosine triphosphate (ATP) to tyrosine residues of specific protein [7, 8]. Imatinib has been approved by the US Food and Drug Administration (FDA), for the GIST iradication [9, 10]. Neoadjuvant chemotherapy using imatinib is being utilized for GIST with a c-kit mutation [11]. Further, it has been demonstrated that imatinib activated CD8(+) T cells and induced regulatory T cell (T(reg) cell) apoptosis within the tumor by reducing tumor-cell expression of the immunosuppressive enzyme indoleamine 2,3-dioxygenase (Ido) [12]. Although imatinib improves the prognosis of patients with advanced GIST [9], approximately 50% of patients develop tumor recurrence within 2 years, due to secondary mutations in c-kit at exons 13, 14, 16, 17 or 18 [5].

Sunitinib, a multi-tyrosine kinase inhibitor, has been considered second-line therapy for GIST, is effective against tumors with c-kit exon 9, 13 or 14 mutations [13]. Regorafenib, another tyrosine kinase inhibitor, was approved by the FDA as a third-line therapy for advanced GIST [14], is effective against tumors with c-kit exon 16, 17 or 18 mutations [15, 16, 17, 18, 19, 20]. Clinical trials of other drugs targeting c-kit and PFGFRα are currently ongoing [21, 22, 23]. However, all these inhibitors have limited clinical efficacy. Therefore, new effective therapy is required to improve the prognosis of GIST. To accomplish this goal of precision therapy, individualized treatment of cancer patients, we have established the patient-derived orthotopic xenograft (PDOX) nude mouse model [24].

*Salmonella typhimurium* A1-R (*S. typhimurium* A1-R) is a facultative anaerobe, which was developed in our laboratory. *S. typhimurium* A1-R can grow and replicate in viable as well as necrotic areas of tumors [25]. *S. typhimurium* A1-R is auxotrophic (leu/arg-dependent) [25, 26] but does not mount a continuous infection in normal tissues. It receives sufficient nutritional support from tumor tissue. *S. typhimurium* A1-R replicates in tumor tissues by more than 1,000-fold compared with normal tissues [26]. *S. typhimurium* A1-R has only auxotrophic mutations for leu and arg, and is therefore not over-attenuated. Further, *S. typhimurium* A1-R was selected for increased virulency by *in vivo* tumor passage [25]. Previously, we reported that tumor-targeting *S. typhimurium* A1-R was effective against many types of PDOX models including melanoma [25, 27, 28, 29], sarcoma [30, 31, 32, 33, 34] and pancreatic cancer [35, 36].

In this manuscript, we evaluated the efficacy of *S. typhimurium* A1-R against the imatinib-resistant GIST PDOX model [37].

## 2 Materials and methods

### 2.1 Animals

In the present study, 4–6 weeks old, athymic *nu/nu* male nude mice (AntiCancer, Inc., San Diego, CA), were utilized. All experimental protocols and data were collected as previously described [27, 28, 29, 30, 31, 32, 33, 34, 35, 36, 37]. All mice were housed in a barrier facility on a high efficiency particulate arrestance (HEPA)-filtered rack under standard 12-hour light-dark cycles conditions. Mice were fed an autoclaved laboratory rodent diet. Anesthesia and analgesics were applied for all surgical experiments. A ketamine mixture (a 0.02 ml solution of 20 mg/kg ketamine, 15.2 mg/kg xylazine, and 0.48 mg/kg acepromazine maleate) was utilized subcutaneously for all mice. The animals were observed carefully during surgery to maintain adequate depth of anesthesia. The animals were monitored daily and humanely sacrificed by CO_2_ inhalation when they met the following criteria: severe tumor burden (more than 20 mm in diameter), prostration, significant body weight loss, difficulty breathing, rotational motion, and body temperature drop.

### 2.2 Establishment of the GIST PDOX model

**Fig. 1:**
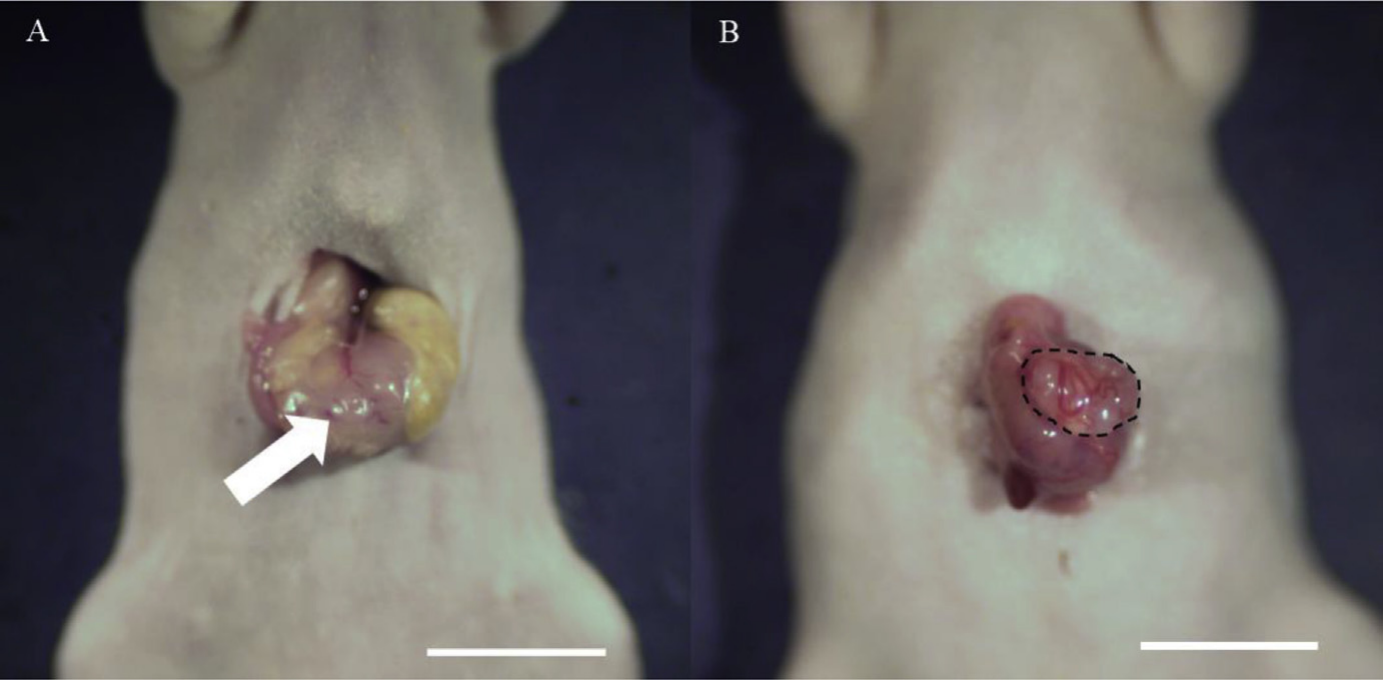
Surgical orthotopic implantation (SOI). A: The stomach of a nude mouse under ketamine anesthesia was gently exteriorized through an abdominal incision and a small GIST fragment was implanted on the anterior gastric wall after slight tearing of the serosa (white arrow). Scale bar: 10 mm. B: The GIST PDOX model was observed after 6 weeks. The area surrounded by black broken lines indicates the established tumor.

The patient received primary surgery in the Department of Surgery, University of California, Los Angeles (UCLA). All experimental protocol and data collection were as described [27, 28, 29, 30, 31, 32, 33, 34, 35, 36, 37]. The patient recurred and received imatinib as neoadjuvant chemotherapy to make the tumor easier to resect and to reduce the chances of rupture. Curative surgery for recurrence was performed and resected fresh tumor was brought to AntiCancer Inc. from the UCLA Hospital. The tumor has mutations in c-kit in exons 11 and 17. The GIST tumor was initially established subcutaneously in nude mice. Surgical orthotopic implantation to the anterior gastric wall of nude mice was subsequently performed to establish the GIST PDOX model using tumor tissue previously grown subcutaneously (Fig. 1-A). Laparotomy was performed to measure the established tumor size when the tumor was palpable over the skin (Fig. 1-B). All experimental protocols provided in this manuscript are based on our previous publications [27, 28, 29, 30, 31, 32, 33, 34, 35, 36, 37, 38, 39, 40].

### 2.3 Preparation and administration of *S. typhimurium* A1-R

All experimental protocols and data were collected as previously described [27, 28, 29, 30, 31, 32, 33, 34, 35, 36, 37]. Green fluorescent protein (GFP)-expressing *S. typhimurium* A1-R bacteria (AntiCancer Inc.,) were grown in LB medium (Fisher Sci., Hanover Park, IL, USA) and then diluted 1:10 in LB medium. Bacteria were harvested at late-log phase, washed twice with PBS, then diluted in phosphate-buffered saline (PBS) to 5 × 10^8^ colony-forming units (CFU)/ml. *S. typhimurium* A1-R (5 × 10^7^ CFU) in 100 μl PBS was injected i.v. in each mouse [38].

### 2.4 Treatment protocol for the GIST PDOX model

**Fig. 2:**
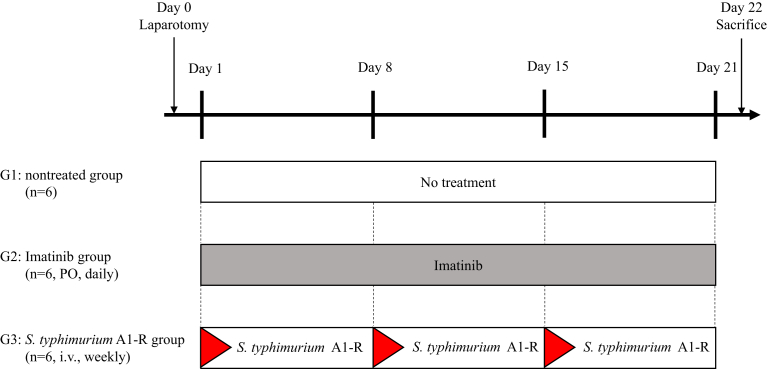
Treatment protocol. G1: untreated group; G2: imatinib (50 mg/kg, oral administration [p.o.], daily, 3 weeks); G3: *S. typhimurium* A1-R (100 CFU/body, i.v., weekly, 3 weeks). Each group consisted of 6 mice. All mice were sacrificed on day 22.

All experimental protocols and data were collected as previously described [27, 28, 29, 30, 31, 32, 33, 34, 35, 36, 37]. The PDOX models were randomized into 3 groups described below when tumor volume reached 60 mm^3^; G1: untreated group; G2: imatinib (50 mg/kg, oral administration [p.o.], daily, 3 weeks); G3: *S. typhimurium* A1-R (5 × 10^7^ CFU/body, i.v., weekly, 3 weeks) (Fig. 2). Dosages of imatinib and *S. typhimurium* A1-R were determined from published reports [32, 37]. Tumor volume was evaluated on day 0 and day 22 by laparotomy with the following formula: tumor volume (mm^3^) = length (mm) × width (mm) × width (mm) × 1/2. Body weight was measured twice a week. All mice were sacrificed on day 22.

### 2.5 Imaging of *S. typhimurium* A1-R

The FV1000 confocal microscope (Olympus, Tokyo, Japan) was used to visualize *S. typhimurium* A1-R-GFP in the GIST PDOX tumor by GFP fluorescence. Fluorescence images were obtained with the 20×/0.50 UPlan FLN and 40×/1.3 oil Olympus UPLAN FLN objectives [39].

### 2.6 Histological examination

10% formalin fixed, paraffin-embedded tissue sections (5 μm) were deparaffinized in xylene and rehydrated in an ethanol series. Hematoxylin and eosin (H&E) staining was done according to standard protocols. Histological examination was observed with a BHS system microscope (Olympus Corp., Tokyo, Japan).

### 2.7 Statistical analysis

All statistical analyses were performed with the Statistical Package for the Social Sciences for Windows software version 22.0 (IBM Corp., Armonk, NY, USA). Significant differences for comparisons of intragroup were determined using one-way ANOVA followed by Tukey post hoc pairwise tests. Bar graphs show mean values and error bars express ± standard deviation. A probability value of *P* < 0.05 was considered as statistical significant.

### 2.8 Ethical considerations

All animal experiments were done at AntiCancer Inc. with an Institutional Animal Care and Use Committee (IACUC)-protocol solely approved for present study and following the principles and procedures defined in the National Institutes of Health (NIH) Guide for the Care and Use of Animals under Assurance Number A3873-1. Informed consent was obtained from the patient, and this study was approved by the Institutional Review Board of UCLA (IRB #10-001857).

## 3 Results

### 3.1 Efficacy of *S. typhimurium* A1-R and imatinib on the GIST PDOX tumor growth

**Fig. 3:**
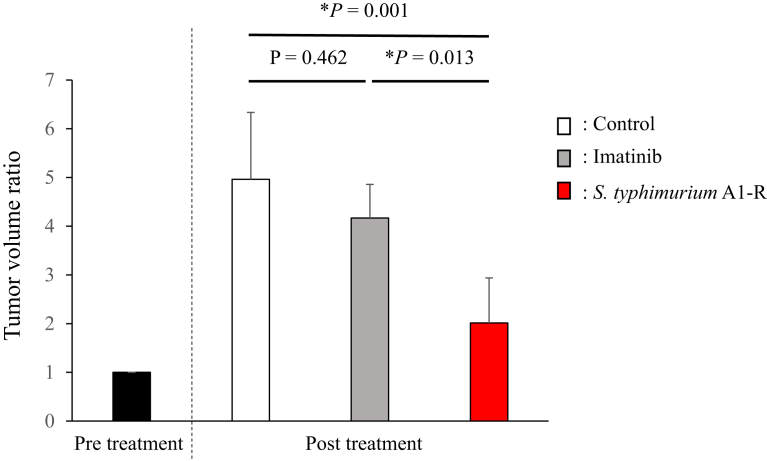
Tumor volume ratio. Bar graphs show the tumor volume ratio (post-treatment volume/pre-treatment volume). *S. typhimurium* A1-R suppressed tumor growth significantly compared to the untreated group (*P* = 0.001). There was also a significant difference between the *S. typhimurium* A1-R group and the imatinib group (*P* = 0.013). Imatinib did not show significant efficacy compared to the untreated control (*P* = 0.462). N = 6 mice/group. Error bars: ±SD.

Imatinib is first line treatment for GIST. Secondary mutations in c-kit have been shown to be the prime cause of resistance to imatinib in GIST. A few studies have demonstrated that GIST patients respond better to imatinib if their tumors contain c-kit mutations in exon 11 compared to exon 9 [5, 41, 42, 43]. Despite the clinical success of imatinib, several studies have attributed the development of acquired resistance of GIST to imatinib. In addition, second and third-generation c-KIT inhibitors were not able to completely overcome imatinib resistance in GIST. Therefore, a better treatment option is urgently needed. To address this issue, we tested the efficacy of tumor-targeting *S. typhimurium* A1-R compared to imatinib on the GIST PDOX. We established the GIST PDOX model using surgical orthotopic implantation in the anterior gastric wall (Fig. 1). The schematic treatment design is shown in Fig. 2. Three weeks after *S. typhimurium* A1-R injection, tumor volumes in each group were weighted. The estimated tumor volume ratio (day 22/day 0) is provided in Fig. 3. We found that *S. typhimurium* A1-R reduced the GIST PDOX tumor growth significantly in contrast to the untreated group (*P* = 0.001). The efficacy of *S. typhimurium* A1-R was much greater than imatinib (*P* = 0.013). However, imatinib treatment did not show significant efficacy in contrast to the untreated group (*P* = 0.462). The final tumor volume ratios (day 22/day 0) were as follows: untreated group (G1) (4.96 ± 1.37); imatinib group (G2) (4.17 ± 0.69); *S. typhimurium* A1-R group (G3) (2.01 ± 0.93).

### 3.2 Effect of imatinib and *S. typhimurium* A1-R treatment on body weight

**Fig. 4:**
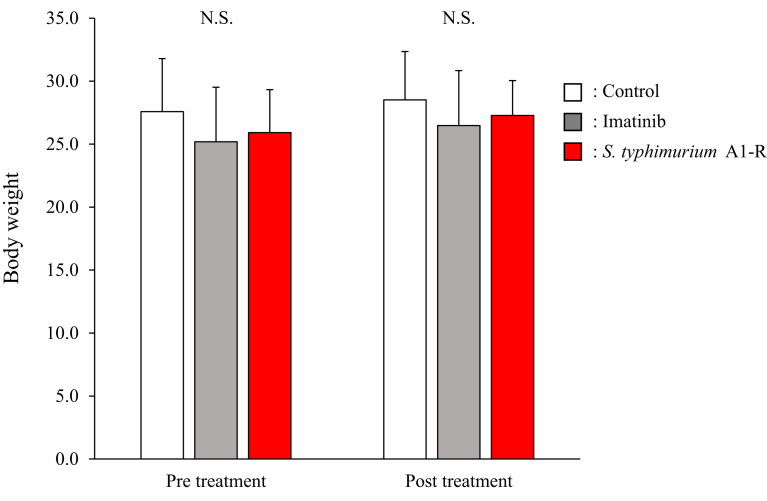
Body weight of each group. Bar graphs show pre-treatment and post-treatment body weight of the GIST PDOX mice treated with each drug. There was no significant difference between any group. N = 6 mice/group. Error bars: ±SD.

The body weight of the GIST PDOX mice was measured pre-treatment and post-treatment either with imatinib or *S. typhimurium* A1-R. We did not find any significant difference in body weight between the three groups (Fig. 4).

### 3.3 Imaging of tumor-targeting *S. typhimurium* A1-R in the GIST PDOX

**Fig. 5:**
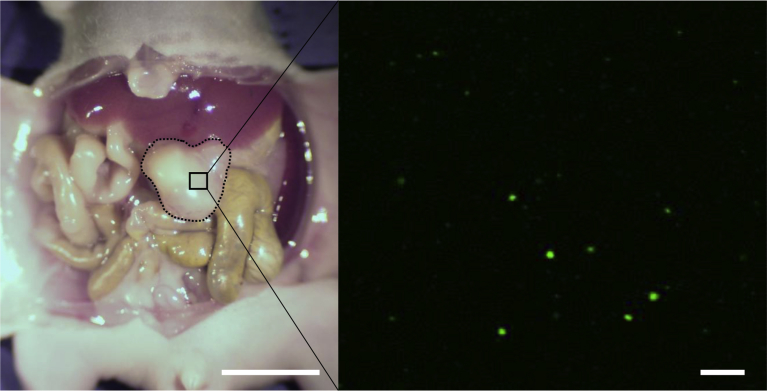
Fluorescence imaging of *S. typhimurium* A1-R-GFP targeting the GIST PDOX model. Broken black lines indicate tumor treated with *S. typhimurium* A1-R which was visualized by confocal imaging with the FV1000 (Olympus, Tokyo, Japan). Left scale bar: 10 mm; Right scale bar: 20 μm.

After intravenous (i.v.) administration of *S. typhimurium* A1-R for three weeks, the accumulation of the GFP-expressing *S. typhimurium* A1-R in the GIST PDOX tumor was observed using confocal microscopy (Fig. 5).

### 3.4 Effect of treatment on size and tumor histology

**Fig. 6:**
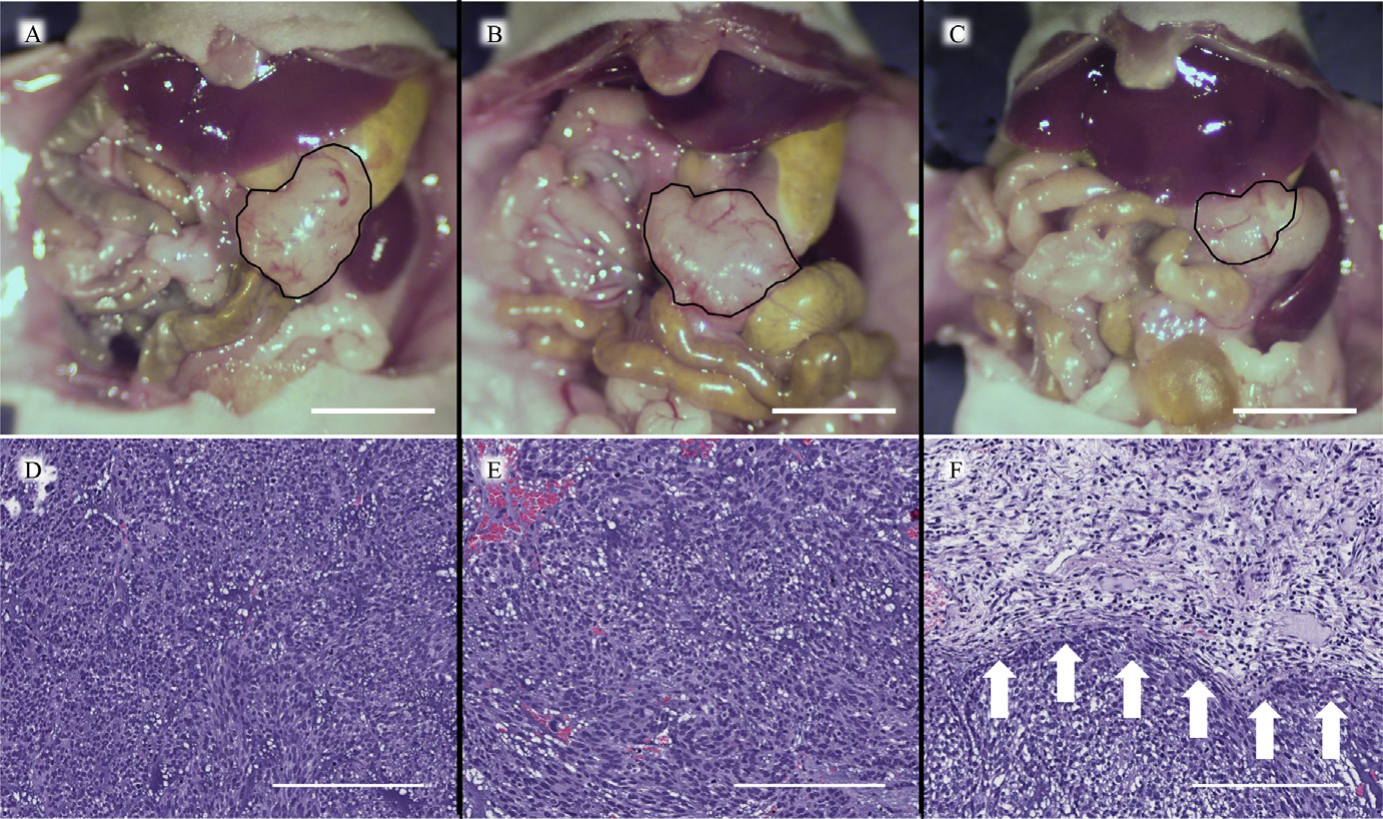
Representative laparotomy images of each group on day 22 (A–C). The area surrounded by the black line indicates tumor. A. Control group. B. Imatinib group. C. *S. typhimurium* A1-R group. Scale bars: 10 mm. Histology (D–F). D. Hematoxylin and eosin (H&E) staining of the untreated PDOX tumor. E. H&E staining of the imatinib-treated PDOX tumor. F. H&E staining of the *S. typhimurium* A1-R-treated PDOX tumor. Necrosis was observed (white arrows). Scale bars: 200 μm.

Laparotomy images at post-treatment on day 22 (Fig. 6A–C) showed that *S. typhimurium* A1-R-treated mice had reduced tumor volume (Fig. 6C) compared to control (Fig. 6A) and imatinib (Fig. 6B) groups. High-power photomicrographs of tumor sections are provided with hematoxylin and eosin (H&E) staining. H&E staining of paraffin-embedded tumor sections did not contain necrotic areas in the control group and imatinib group (Fig. 6A, B). However, *S. typhimurium* A1-R caused extensive necrosis (Fig. 6C).

## 4 Discussion

Our present study demonstrates the power of the PDOX model to establish novel efficacious therapy for resistant cancer. To fulfill the goal of precision personalized oncology, our laboratory established the PDOX nude mouse model using the technique of surgical orthotopic implantation (SOI), including pancreatic [40, 41, 42, 43, 44, 45], breast [46], ovarian [47], lung [48], cervical [49], colon [50, 51, 52], and stomach cancer [53], sarcoma [34, 35, 54, 55, 56, 57, 58, 59, 60, 61], and melanoma [30, 31, 32, 62, 63].

Accumulative evidence suggests the effectiveness of imatinib in both the adjuvant and metastatic GISTs in patients with *KIT* or *PDGFRA* mutations [64, 65, 66, 67]. However, imatinib was not effective in all patients, because it was found that GIST patients with mutations in the exon 9 of the *KIT* gene have lower effectiveness compared to mutations in exon 11 [41, 67, 68].

Since exon-11-mutant KIT GIST is heterogeneous, it was advisable to plan treatment strategies based on individual mutant cohorts [68]. Imatinib can improve the prognosis of advanced GIST patients [68, 69]. However, almost all such cases become resistant to these drugs due to secondary mutations in c-kit or PDGFRα [68, 69]. The present case also had a secondary mutation in exon 17 and failed neoadjuvant therapy using imatinib. For these reasons, the development of new effective therapy for TKI-resistant GIST remains a major challenge.

*S. typhimurium* A1-R may be a general therapeutic for cancer. *S. typhimurium* A1-R is auxotrophic for leu–arg, which prevents it from mounting a continuous infection in normal tissues. *S. typhimurium* A1-R inhibited or eradicated primary and metastatic tumors as monotherapy in nude-mouse models of major cancers [70, 71], including prostate [72], breast [38, 46, 73, 74], lung [75], pancreatic [36, 37, 76, 77, 78], ovarian [79, 80], stomach [81], and cervical cancer [82], glioma [83, 84], as well as sarcoma [35, 54], including osteosarcoma [85, 86], all of which are highly aggressive tumor models. Several different types of bacteria such as *Salmonella, Listeria, Escherichia*, and *Clostridium* have been reported to either target or destroy solid tumors. Among these bacteria, several strains of *Salmonella* even colonize solid tumors and display antitumor immunity [87]. Thus, tumor-targeting bacteria have great potential in curing solid tumors [88, 89, 90, 91, 92, 93, 94].

In the present case, *S. typhimurium* A1-R was effective in the GIST PDOX model with a secondary mutation in exon 17, suggesting that *S. typhimurium* A1-R be developed clinically for TKI-resistant GIST patients. *S. typhimurium* A1-R showed significant efficacy in an imatinib-resistant GIST PDOX model with secondary mutations in the c-kit gene. *S. typhimurium* A1-R decoys quiescent tumor cells from G_0_/G_1_ to S/G_2_/M phase, which makes cancer cells sensitive to chemotherapy [82]. We previously reported that *S. typhimurium* A1-R in this way overcomes the resistance to chemotherapy [30, 34]. Thus, the combination of *S. typhimurium* A1-R and imatinib to overcome the resistance to imatinib is warranted going forward. Further, *S. typhimurium* A1-R efficacy on other subtypes of GIST in PDOX models should be investigated in the future.

Future experiments are needed to understand the molecular mechanism by which *S. typhimurium* A1-R could overcome the imatinib-resistant GIST PDOX model. This would be crucial for designing effective treatment regimens. Kim et al. [94] reported that tumor colonization by *S. typhimurium* defective in ppGpp synthesis *(ΔppGpp Salmonellae)* resulted in a significant increase in the level of interleukin (IL)-1β and tumor necrosis factor-α (TNF-α) within the tumor mass, specifically during the tumor-suppression stage. Their results suggest that IL-1β and TNF-α play crucial roles in *Salmonella*-mediated cancer therapy. We also found accumulation of *S. typhimurium* A1-R within the tumor site and increased necrosis in our GIST PDOX. It is also reported that systematic injection of IL2-expressing *S. typhimurium* reduced angiogenesis and increased necrosis within tumor tissues [95]. We believe that one of above these mechanisms may be effective in our model system. Long-term followup and more patient data will be needed to further validate our findings.

Zhao et al. [71] reported that *S. typhimurium* A1-R directly targets and kills cancer cells. The present study reflects this property. It is known that bacteria usually can be efficiently eliminated by the immune system in immunocompetent mice. However, after tumor necrosis high concentrations of nutrients such as purines [96] and lack of immunologic surveillance promote the growth and survival of bacteria at the tumor site [96]. Li et al. [97] using immune-deficient nude mice reported that *S. typhimurium* strain SL7207 was enriched in the tumor and inhibited tumor growth. These results suggest that systemic *S. typhimurium* may be eliminated by mechanisms other than the immune system. Using nude-mouse models which are T-cell deficient, it has been shown that *S. typhimurium* A1-R destroys tumor blood vessels [76]. In addition, an increase in TNF-α in blood and a high influx of blood into tumors by vascular disruption resulted in influx of bacteria into the tumor together with the blood [98]. Since these and our studies used immunodeficient mice, it will be crucial to test the anticancer efficacy of *S. typhimurium* A1-R in an immunocompetent mice. Recently Zhang et al. [99] tested the toxicologic and biodistribution of tumor-targeting *S. typhimurium* A1-R and *S. typhimurium* VNP20009 in a syngeneic tumor model growing in immunocompetent BALB/c mice. They found a safe dose and schedule of *S. typhimurium* A1-R administration in BALB/c mice that is efficacious against tumor growth. They found greater antitumor efficacy of *S. typhimurium* A1-R in an immunocompetent mouse model [99]. These results together with present data suggest that *S. typhimurium* A1-R could be a therapeutic option for imatinib-resistant GIST.

## Declarations

### Author contribution statement

Kentaro Miyake: Conceived and designed the experiments; Performed the experiments; Analyzed and interpreted the data; Wrote the paper.

Kei Kawaguchi, Masuyo Miyake, Ming Zhao, Tasuku Kiyuna, Kentaro Igarashi, Zhiying Zhang, Takashi Murakami: Performed the experiments; Analyzed and interpreted the data.

Yunfeng Li, Scott D. Nelson, Michael Bouvet, Irmina Elliott, Tara A. Russell, Arun S. Singh, Yukihiko Hiroshima, Masashi Momiyama, Ryusei Matsuyama, Takashi Chishima, Shree Ram Singh, Itaru Endo, Fritz C. Eilber: Analyzed and interpreted the data.

Robert M. Hoffman: Conceived and designed the experiments; Analyzed and interpreted the data; Wrote the paper.

### Funding statement

This work was supported by a grant from Yokohoma City University School of Medicine GUSHINKAI Almuni Association, who paid for the publication fees. They had no role in the research or in the writing of the paper.

### Competing interest statement

The authors declare the following conflict of interests: Kentaro Miyake, Kei Kawaguchi, Masuyo Miyake, Tasuku Kiyuna, Kentaro Igarashi, Zhiying Zhang and Robert M. Hoffman are unsalaried associates of AntiCancer Inc. There are no other competing financial interests.

### Additional information

No additional information is available for this paper.
